# VULGARE ROW-TYPE SIX 5 binds to the promoter of tillering and floral homeotic genes to regulate their expression

**DOI:** 10.1093/plphys/kiaf309

**Published:** 2025-07-17

**Authors:** Ton Winkelmolen, Pierangela Colleoni, Matthew J Moscou, Parastoo Hoseinzadeh, Klaus Oldach, Ralf Christian Schmidt, Richard G H Immink, G Wilma van Esse

**Affiliations:** Laboratory of Molecular Biology, Wageningen University and Research, Wageningen 6708 PB, The Netherlands; Laboratory of Molecular Biology, Wageningen University and Research, Wageningen 6708 PB, The Netherlands; The Sainsbury Laboratory, Norwich Research Park, Norwich NR47 UH, UK; KWS SAAT SE & Co. KGaA, 37574 Einbeck, Germany; KWS SAAT SE & Co. KGaA, 37574 Einbeck, Germany; BASF Belgium Coordination Center CommV, Technologiepark 101, 9052 Gent Zwijnaarde, Belgium; Laboratory of Molecular Biology, Wageningen University and Research, Wageningen 6708 PB, The Netherlands; Bioscience, Wageningen Plant Research, Wageningen University and Research, Wageningen 6708 PB, The Netherlands; Laboratory of Molecular Biology, Wageningen University and Research, Wageningen 6708 PB, The Netherlands

## Abstract

Variation in shoot architecture, or tillering, is an important adaptive trait targeted during the domestication of crops. A well-known regulatory factor in shoot architecture is TEOSINTE BRANCHED 1 (TB1). TB1 and its orthologs have a conserved function in integrating environmental signals to regulate axillary branching or tillering in cereals. The barley (*Hordeum* vulgare) ortholog of TB1, VULGARE ROW-TYPE SIX 5 (VRS5), regulates tillering and is involved in regulating row-type by inhibiting lateral spikelet development. These discoveries predominantly come from genetic studies; however, how VRS5 regulates these processes on a molecular level remains largely unknown. By combining transcriptome analysis between the vrs5 mutant and the wild-type at different developmental stages and DAP-sequencing to locate the genome-wide DNA binding sites of VRS5, we identified bona fide targets of VRS5. We found that VRS5 targets abscisic acid-related genes, potentially to inhibit tillering in a conserved way. Later in inflorescence development, VRS5 also targets row-type gene *VRS1* and several known floral development genes, such as MIKCc-type MADS-box genes. This study identifies several genes for mutational analysis, representing a selection of bona fide targets that will contribute to a deeper understanding of the VRS5 network and its role in shaping barley development.

## Teaser

VULGARE ROW-TYPE SIX 5 directly regulates tillering and lateral spikelet development.

## Introduction

Variation in shoot architecture is one of the most important adaptive traits that have been targeted during crop domestication (Doebley et al. 2006). In cereals like barley and wheat (*Triticum aestivum*), the aboveground architecture is determined by the number of branches, called tillers, and inflorescence (spike) architecture (Dixon et al. 2022). Tillers are formed during vegetative growth of the shoot apical meristem, which produces successive leaves with (vegetative) axillary meristems (AM) in the leaf axil. The regulation of tillering usually happens after AM initiation, where the developing AM can enter a stage of dormancy as a bud (Hussien et al. 2014; Shang et al. 2021; Riaz et al. 2023). The trigger to develop a bud into a tiller, or to remain dormant, depends on both internal and external cues (Wang et al. 2018; Shaaf et al. 2019; Dixon et al. 2022).

One of the key players that are involved in the control of bud outgrowth is TEOSINTE BRANCHED 1 (TB1). TB1 orthologs have been targeted during domestication for their impact on plant architecture and development (Wang et al. 1999; Clark et al. 2004; Martín-Trillo et al. 2011). In maize (*Zea mays*) for example, increased expression of *ZmTB1* contributes to a complete suppression of the lateral tillers and increased crop yield (Doebley et al. 1995; Studer et al. 2011). TB1 is a member of the TEOSINTE BRANCHED 1/CINCINNATA/PROLIFERATING CELL FACTOR (TCP) family. TCP family members regulate gene expression trough DNA binding and act as transcriptional regulators. Recent studies in maize indicate that at the molecular level TB1 directly regulates genes involved in hormone signaling, including abscisic acid (ABA), gibberellic acid (GA), and jasmonate (JA), and sugar signaling (Dong et al. 2019).

Besides its function in the control of tiller outgrowth, TB1 plays an additional role in orchestrating spike architecture in the *Triticeae*. In wheat, high levels of *TaTB-D1* result in reduced expression of meristem identity genes, thereby promoting paired spikelet formation (Dixon et al. 2018). In barley, the *TB1* ortholog, *VULGARE ROW-TYPE SIX 5* (*VRS5*), which is also known as *INTERMEDIUM-C* (*INT-C*), impacts not only tiller development, but also represses lateral spikelet development. When compared to wild-type lines, *vrs5* (*int-c*) mutants exhibit an increased tiller number at early developmental stages and developed lateral florets of the triple spikelet meristem, leading to a 6-rowed spike phenotype in contrast to a 2-rowed spike in wild-type barley (Ramsay et al. 2011; Liller et al. 2015; Zwirek et al. 2019). In 2-rowed cultivars, the formation of lemma and carpel primordia stagnates in the lateral florets, making them infertile, which means the lateral florets do not form a grain. In contrast, carpel primordia are formed and fully developing in *vrs5* mutants, making them fertile, which means the lateral florets do develop into grains (Zwirek et al. 2019). Taken together, functional VRS5 in 2-rowed cultivars prevents the formation of carpel primordia in the lateral spikelets. In line with the mode of action of VRS5 in tiller and lateral spikelet development, *VRS5* is expressed in the tiller buds and the lateral spikelet primordia of developing inflorescences (Thiel et al. 2021; Wang et al. 2022).

Despite the key role of VRS5 in orchestrating tillering and lateral spikelet development in barley, the molecular mode of action remains poorly understood. To gain a better understanding of the regulatory network by which VRS5 controls both tiller and inflorescence development, we have profiled transcriptional changes associated with altered development in the *vrs5* mutant. This comprehensive analysis enabled us to define the *vrs5*-related transcriptional changes over development. In combination with a genome-wide transcription factor (TF) binding site mapping using DNA affinity purification (DNA affinity purification and sequencing (DAP-seq)), we identified bona fide VRS5 targets. Among these bona fide targets are floral homeotic and meristem identity genes. In addition, our transcriptional profiling combined with DAP-seq using VRS5 as bait, reveals an evolutionarily conserved and divergent role of VRS5 in the regulation of tiller and grain development. Taken together, this study shows a comprehensive view of the core molecular network downstream of VRS5 and provides candidate genes that can be exploited in the regulation of tillering and inflorescence architecture in cereals.

## Results

### VRS5 regulates both tillering and row-type

**Figure 1.**
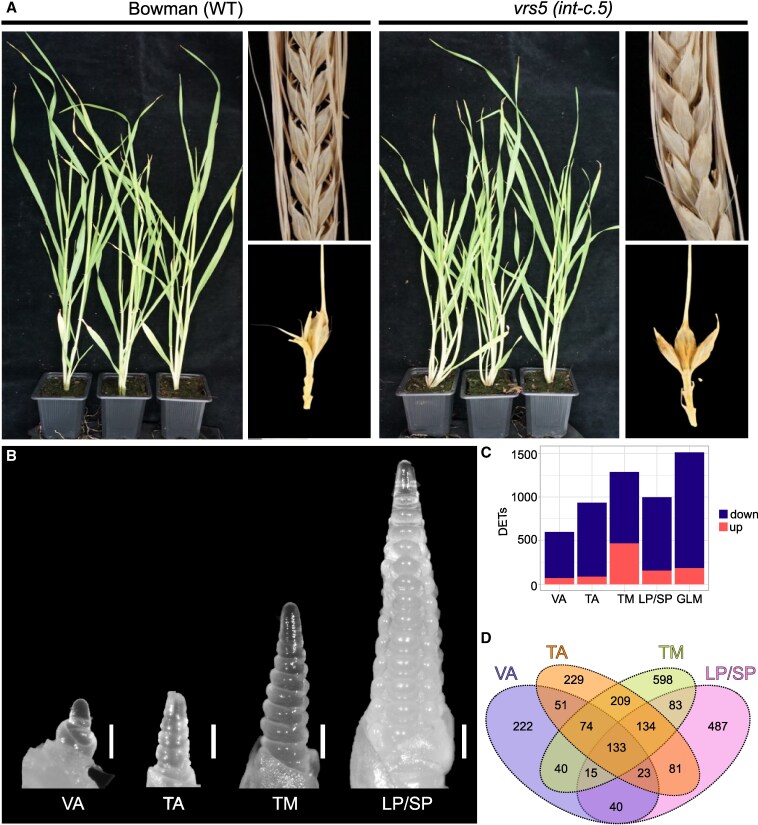
VRS5 regulates both tiller number and lateral spikelet development in barley. **A)** Four-week-old plants, dried spike, and spikelet triplet of wild-type (WT) (Bowman) and vrs5 (int-c.5), respectively. The lateral spikelets of WT have not developed, whereas the lateral spikelets of vrs5 have developed into grains. **B)** Binocular images of developing inflorescence meristems representing the 4 stages used for transcriptome data, namely: VA, TA, TM, and LP/SP. Images were digitally extracted for comparison. The white bar represents a length of 200 µm. **C)** Bar graph showing number of DETs per developmental stage (VA, TA, TM, and LP/SP) and throughout development using a GLM. The direction of differential expression in vrs5 compared to Bowman is shown in blue (down) and red (up). For each developmental stage, 3 biological replicates were used. **D)** Venn-diagram showing overlap of DETs at different developmental stages (VA, TA, TM, and LP/SP).

Previous mutant analysis revealed a role for *VRS5* in tillering and in determination of the row-type in the spikelet (Ramsay et al. 2011; Liller et al. 2015; Wang et al. 2022). Significant differences in tillering between *vrs5* and Bowman were observed already early in development (Fig. 1A and B; Supplementary Fig. S1A), corroborating previous reports (Ramsay et al. 2011; Liller et al. 2015; Zwirek et al. 2019; Wang et al. 2022) . To identify genes regulated by VRS5, we performed a transcriptome analysis on shoot apexes of WT (*Bowman*) and *vrs5* (*int-c.5*) mutants at 4 distinct developmental stages. This approach enabled us to investigate the role of VRS5 across early and later stages of development, including tiller bud outgrowth and inflorescence development. The vegetative apex (VA) and transition apex (TA) were sampled as 2 early developmental stages (Fig. 1B). These samples include the entire crown, which comprises of the (vegetative) meristem and tiller buds. To identify differentially regulated genes early in spikelet and floral organ development, we isolated RNA for transcriptome profiling from developing inflorescences at the triple mound (TM), and lemma primordia and stamen primordia stage (LP/SP), respectively. At these stages, the central and lateral floret primordia are initiated, and the first floral organ primordia are formed (Fig. 1B; Supplementary Fig. S1B).

From the generated transcriptomes, principal component analysis (PCA) of all expressed transcripts showed that developmental stage (PC1) and the genotype (PC2) contribute the most to the observed variation (Supplementary Fig. S2). To gain further insight into the differentially expressed genes between *vrs5* and Bowman, we compared the transcriptomes in 2 different ways, namely at each developmental stage specifically (VA, TA, TM, and LP/SP), and throughout development using a generalized linear model (GLM) taking all 4 stages into account (Supplementary Table S1). When comparing differentially expressed transcripts (DETs) at each developmental stage, most DETs (1286) were found at TM, whereas at VA the least DETs (598) were found (Fig. 1C). Comparing the overlap in DETs in the different stages revealed that 133 transcripts were different between *vrs5* and Bowman in all developmental stages (Fig. 1D). Of the total amount of DETs found, the majority (∼75%) was downregulated in *vrs5* compared to Bowman, indicating that VRS5 mainly acts as a transcriptional activator (Fig. 1).

### Transcriptional profiling of genes differentially regulated in *vrs5*

**Figure 2.**
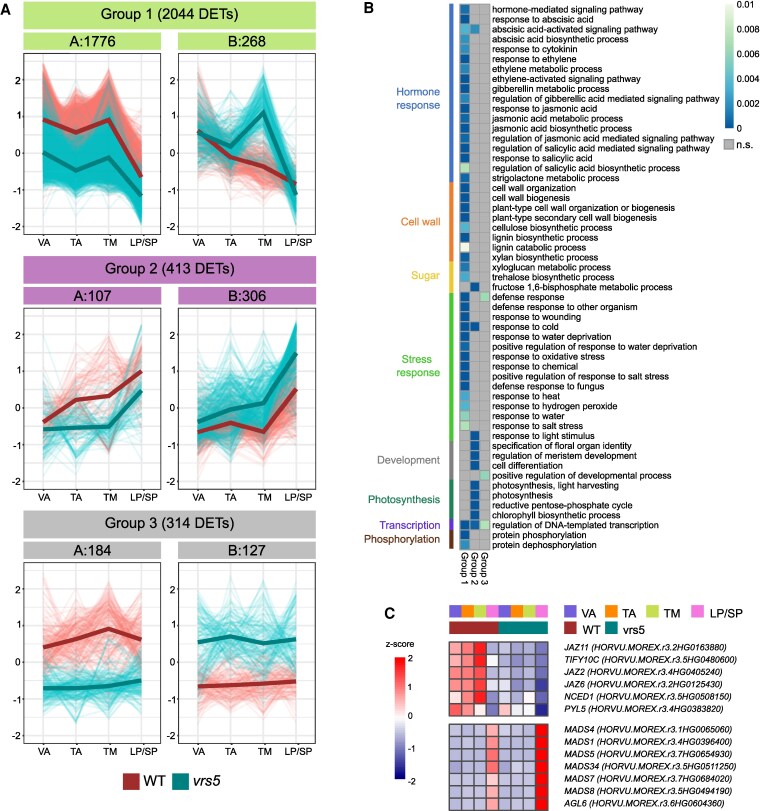
Transcriptional profiling of VRS5 shows changes in gene expression during development. **A)** Line graphs of z-scores of DETs in 3 main coexpression cluster groups: Group 1 showing a downward expression trend, Group 2 showing an upward expression trend, and Group 3 showing no differential expression throughout development. Lines are colored red for wild-type (WT) and cyan for vrs5. Each plot has the developmental stages used for RNA-seq on the *x* axis: VA, TA, TM, and LP/SP. For each developmental stage, 3 biological replicates were used. The groups are divided in A (lower expression in vrs5 compared to WT), and B (higher expression in vrs5 compared to WT). **B)** Enriched GO-terms for biological processes per cluster group where the more blue means a lower *P*-value. A gray color means that the GO-term is not enriched in that cluster group (*P* > 0.01) or the number of DETs belonging to that GO-term are lower than 3. **C)** Heatmap of z-scores of expression of a selection of DETs at each developmental stage: VA, TA, TM, and LP/SP, for WT and vrs5.

To gain a deeper understanding of differential gene expression between the wild-type and *vrs5* mutant over development, a coexpression clustering was performed using 2,872 DETs that were differentially regulated in one or more stages, or independent of development. This yielded 39 clusters, which were subdivided into 3 main groups based on their expression pattern (Fig. 2A; Supplementary Fig. S3; Supplementary Table S2): Group 1 represents genes that are highly expressed at VA and show a downward expression trend toward LP/SP stage; Group 2 represents genes that are lowly expressed at VA, and show an upward expression trend toward LP/SP stage; Group 3 represents genes that have a similar expression over the developmental stages (Fig. 2A). Group 1 has clearly the most DETs, from which the majority, are in Group 1A (1,776, ∼62%), which consists of DETs that go down in expression over time and have lower expression in *vrs5* (Fig. 2A).

To gain insight in the downstream processes, VRS5 regulates during development, gene ontology (GO)-term enrichment was performed on the expression groups. Genes that are highly expressed during VA (Group 1), and differentially regulated in *vrs5* when compared to wild-type lines are significantly enriched for GO-terms related to protein phosphorylation and phytohormone response and signaling (Fig. 2B). DETs in Group 1, annotated with these GO-terms include several JA ZIM-domain containing proteins (JAZ), involved in jasmonic acid (JA) signaling: *JAZ11* (*HORVU.MOREX.r3.2HG0163880*)*, TIFY10C* (*HORVU.MOREX.r3.5HG0480600*)*, JAZ2* (*HORVU.MOREX.r3.4HG0405240*), and *JAZ6* (*HORVU.MOREX.r3.2HG0125430*), which are all downregulated in *vrs5* (Fig. 2C; Supplementary Fig. S4). Genes involved in ABA biosynthesis and signaling are also enriched in Group 1, like *NCED1* (*HORVU.MOREX.r3.5HG0508150*) a 9-cis-epoxycarotenoid dioxygenase (NCED) involved in ABA biosynthesis and *PYL5* (*HORVU.MOREX.r3.4HG0383820*), an ABA receptor. Orthologs of these genes are also differentially expressed in maize *tb1* mutants compared to WT (Dong et al. 2019), suggesting a conserved regulation by the TF proteins encoded by the orthologous genes *ZmTB1* and *VRS5*. Next to GO-terms involved in phytohormone signaling, GO-terms involved in stress response are highly enriched early in development.

Genes in Group 2 are highly expressed at LP/SP stage when compared to VA and TA stages. GO-overrepresentation analysis of DETs between Bowman and *vrs5* lines showed that in this group genes that are involved in “regulation of meristem development,” “plant organ development,” and “cell differentiation” are overrepresented (Fig. 2B). This may reflect the subsequent development of lateral florets in *vrs5* in contrast to their repression in WT. In line with this, we also identified floral homeotic genes among the upregulated genes including *MADS4* (*HORVU.MOREX.r3.1HG0065060*), homologous to *Arabidopsis PISTILLATA*; and all identified E-class MIKCc MADS box genes in barley: *MADS1* (*HORVU.MOREX.r3.4HG0396400*), *MADS5* (*HORVU.MOREX.r3.7HG0654930*)*, MADS34* (*HORVU.MOREX.r3.5HG0511250*)*, MADS7* (*HORVU.MOREX.r3.7HG0684020*), and *MADS8* (*HORVU.MOREX.r3.5HG0494190*) (Fig. 2C; Supplementary Fig. S4) (Kuijer et al. 2021). Also other MIKCc MADS box genes were differentially expressed in *vrs5*, including *AGL6* (*HORVU.MOREX.r3.6HG0604360*), orthologous to rice *MADS6,* and wheat *AGL6*, which is also upregulated in *vrs5* (Fig. 2C) (Kuijer et al. 2021). In barley and wheat, *agl6* mutants are infertile due to defective pistils. Moreover, *TaAGL6* was shown to act upstream of several floral homeotic genes (Kong et al. 2022; Shen et al. 2024).

Our results show that VRS5 during early, tiller developmental stages, is targeting among others, phytohormone and stress signaling associated genes. In contrast, during inflorescence development mainly genes involved in meristem development and floral organ identity are differentially regulated in the absence of VRS5. Together, our results suggest that VRS5 regulates different genes and processes depending on the specific developmental stage.

### Genome-wide identification of the bona fide VRS5 targets

**Figure 3.**
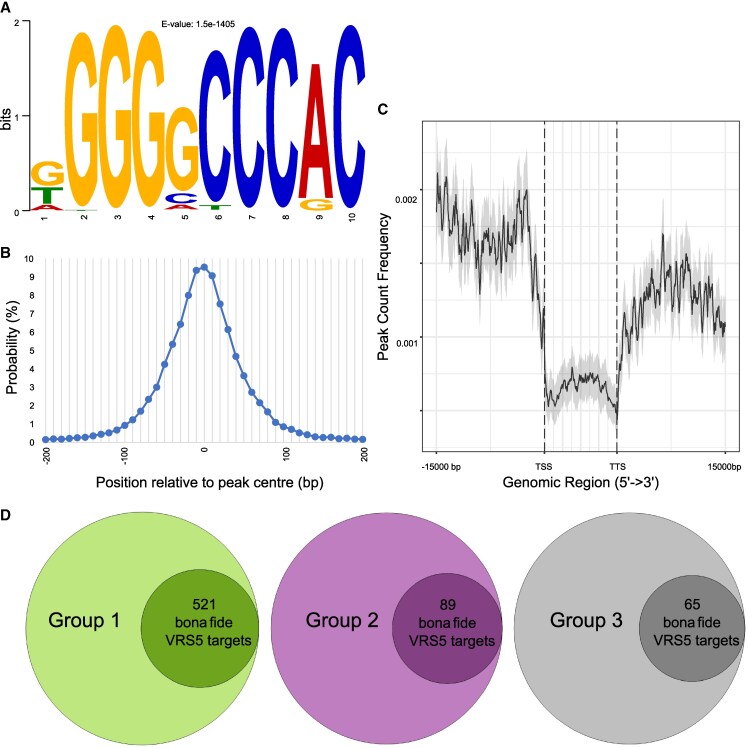
DAP-seq of VRS5 on barley genomic DNA library. **A)** Enriched sequence logo in the VRS5 bound DAP-seq peaks, identified using STREME. **B)** The position of the motif shown in A relative to the center of the VRS5 DAP-seq peak as a probability percentage of bins of 10 bp. **C)** Frequency distribution plot of the VRS5 peaks 15 kb upstream of the Transcription Start Site and 15 kb downstream of TTS along expressed genes in the developmental stages sampled. **D)** Differentially regulated transcripts (DETs) per group that have a VRS5 binding peak within 15 kb of their open reading frame in the DAP-seq experiment. *n* = 6 DAP-seq libraries for VRS5 with *n* = 6 controls.

The transcriptome profiling showed that there are differences in the differentially regulated genes between early, tillering, versus later inflorescence developmental stages. As a TF, VRS5 is expected to regulate the expression of downstream target genes by directly binding to the (distal) promoter region of these genes. As key regulator, VRS5 may only bind to a limited number of downstream genes to trigger a wider transcriptional response. Alternatively, VRS5 may directly activate the majority of the genes identified as DET. To gain more insight into which genomic regions are bound by VRS5, and which of these are within a promoter region of a gene, we performed a DNA-affinity purification followed by genome-wide sequencing (DAP-seq) (Bartlett et al. 2017). To construct our DAP-seq libraries, we used DNA isolated from barley developing inflorescences (Waddington stage ∼4). VRS5 fused to a FLAG-tag was used as bait, while GFP, which is expected not to bind DNA, fused to a FLAG-tag was used as negative control. Regions in the genome enriched for VRS5 binding compared to GFP are called peaks and considered genomic VRS5 binding sites. Only the peaks with an adjusted *P*-value smaller than 1^−10^ were kept, which resulted in 99,567 peaks (Supplementary Table S3). The sequences of all the peaks were analyzed for de novo enriched motifs. The most frequently abundant motif was GGGNCCCAC, found in 72% of the peaks (Fig. 3A). This motif was distributed closely around the peak summit (Fig. 3B), indicating the GGGNCCCAC motif is the primary DNA motif bound by VRS5. This motif resembles a consensus class II TCP-binding motif (GGGNCCAC), and the consensus binding motifs found for homologous TCP TFs in *Arabidopsis* and maize (Kosugi and Ohashi 2002; Gonzalez-Grandio et al. 2017; Dong et al. 2019; van Es et al. 2023). This supports the association of identified peaks as likely genomic VRS5 binding sites.

To assign peaks to putative target genes, we selected a region of 15 kb up and downstream of all predicted barley open reading frames. Within these genomic regions, we found 21% of all identified VRS5-DAP-seq peaks. The other peaks were located outside this region and classified therefore as “Distal intergenic.” Only a few binding sites, 0.1% of the total number of peaks, were identified in intronic regions (Supplementary Table S4). To further understand where the peaks are located in the upstream and downstream region of the gene, we generated a frequency plot of all peaks that were found in genes that are expressed in at least 1 of the VA, TA, TM, or LP/SP stages. Results showed that peaks in expressed genes were mostly found to bind the upstream and downstream region (15 kb) of the gene body which includes the promoter and terminator, rather than the gene body itself (Fig. 3C). By combining the VRS5 binding peaks with the DETs found in our expression analysis between *vrs5* and WT, we identified putative direct downstream targets of VRS5 (bona fide VRS5 targets). We identified DAP peaks corresponding to all 3 coexpression groups (Fig. 3D) with: Group 1 representing genes highly expressed at the VA and downregulated at late reproductive stages, and enriched for ABA and JA hormone pathways; Group 2, representing genes highly expressed during inflorescence development and low expressed during the early developmental stages, genes in this group include key regulators of inflorescence and floral organ development, such as MIKC-type MADS-box genes and the barley *lgm1* candidate; and Group 3, which mainly contains genes over represented in development and defense responses (Fig. 2B). To evaluate if these are equally distributed over the 3 expression groups identified in the coexpression clustering, we compared the percentage of genes that were differentially regulated in the transcriptional profiling, and of which the upstream and/or downstream region was bound by VRS5 within the DAP-seq experiment. Results showed that in all 3 expression groups, the percentage of DETs with a VRS5-DAP-seq peak was similar (∼20–25%). This suggests that VRS5 is directly regulating transcription of its targets throughout the developmental stages tested. Next, we extended our data set that comprises 675 bona fide targets trough reanalysis of publicly available transcriptome data. These set include differentially regulated genes of *vrs5* (*int-c.5*) versus wild-type of the SP and awn primordia (AP) stages (van Esse et al. 2017) and the tiller buds only (Wang et al. 2022). Inclusion of these additional stage resulted in 754 bona fide targets (Supplementary Tables S5–S7). Taken together, our analysis results in the identification of bona fide VRS5 targets.

### VRS5 controls genes involved in inflorescence development

To further explore the role of VRS5 in regulating genes involved in inflorescence development, we focus on the bona fide VRS5 targets identified in Group 2, which comprises DETs that are low at VA and TA and high at the LP/SP stages. We identified several known genes in inflorescence and flower development. For example, a C2H2 zinc finger (*HORVU.MOREX.r3.2HG0170820*), which is identified as candidate for the long glume mutant (*lgm1*) in barley (Zhang et al. 2024b) was significantly upregulated in *vrs5* when compared to wild-type and among the bona fide targets. Its ortholog in wheat is causing awn suppression (DeWitt et al. 2020; Huang et al. 2020). Other targets known in inflorescence and flower development include several MIKC-type MADS-box genes, which have a conserved role in the specification of floral organ identity (Kuijer et al. 2021). Among these are orthologs of floral organ development genes such as: *MADS4* (*HORVU.MOREX.r3.1HG0065060*), *MADS1* (*HORVU.MOREX.r3.4HG0396400*)*, AGL6* (*HORVU.MOREX.r3.6HG0604360*), and *SOC1-like* (*HORVU.MOREX.r3.1HG0054220*) that also contained a DAP-seq peak and were differentially upregulated in *vrs5* mutants when compared to wild-type lines in the RNA-seq (Fig. 2C; Supplementary Table S3).

**Figure 4.**
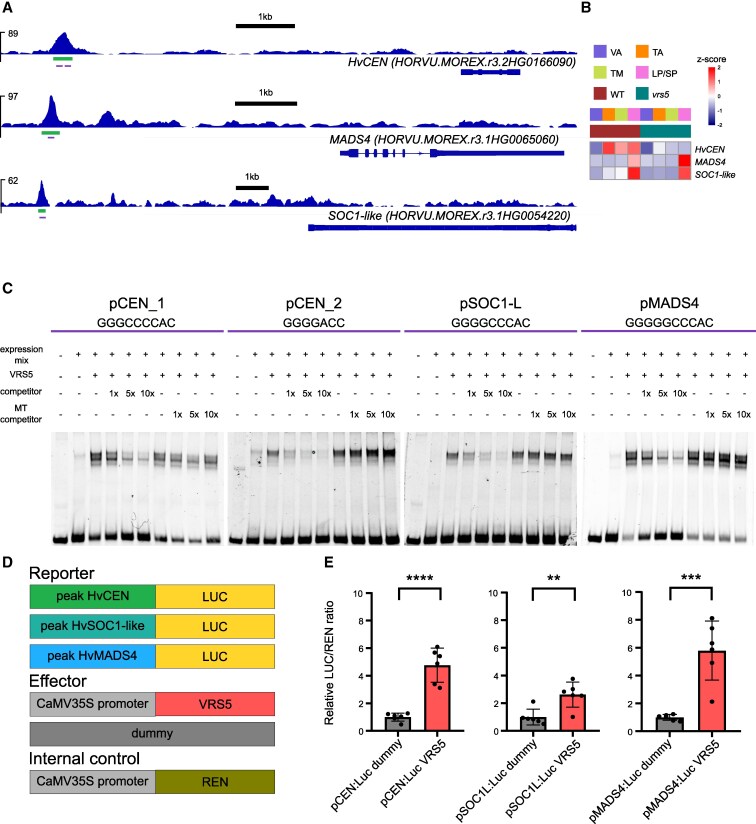
VRS5 binds to regions upstream of genes involved in inflorescence development. **A)** VRS5 DAP-seq peaks identified upstream of HvCEN, *MADS4,* and *SOC1-like*. The green bars represent the peak significant compared to the control. The purple bars mark the VRS5 binding sites within the peaks indicating that: there are two VRS5 binding sites upstream of *HvCEN* (pCEN_1 and pCEN_2); one upstream of *MADS4* (pMADS4); and one upstream of *SOC1-like* (pSOC1-L). **B)** Heatmap of z-scores of expression of a selection of DETs at each developmental stage: VA, TA, TM, and LP/SP, for wild-type (WT) and vrs5. **C)** Confirmation of the VRS5 binding to DAP-seq peaks using EMSAs on the VRS5 binding sites identified upstream of HvCEN (pCEN), MADS4 (pMADS4), and SOC1-like (pSOC1-L). The peak upstream of HvCEN contained 2 putative VRS5 binding sites within the DAP peak, which are indicated as pCEN_1 and pCEN_2. Each probe is ∼80 bp long and the sequence of the TCP binding site is indicated. For each probe, the first lane contains only labeled DNA probe, the second lane contains labeled DNA probe and expression mix without VRS5 plasmid, and the other lanes contain labeled DNA probe and expression mix with VRS5 plasmid. Unlabeled competitor or unlabeled shuffled competitor (MT competitor) is added to indicate lanes in 1×, 5×, and 10× concentrations compared to labeled probe. **D)** Schematic overview of the different reporter and effector combinations used. For the reporter constructs, the VRS5 DAP-peak was cloned before the CDS of Luciferase. **E)** Data show relative LUCCIFERASE (LUC)/RENILLA (REN) ratio in a dual-luciferase assay in *N. benthamiana* leaves. The mean LUC/REN ratios are relative to the mean LUC/REN ratio of the dummy for each reporter. The red bar shows the mean LUC/REN ratio when VRS5 is used as effector. Black dots indicate individual measurements (*n* = 6). Error bars indicate standard deviation. Asterisks designate significant differences of the mean (Student's *t*-test, **: *P* < 0.01, ***: *P* < 0.001, and ****: *P* < 0.0001).

*CENTRORADIALIS* (*HvCEN; HORVU.MOREX.r3.2HG0166090*), is a gene involved in the regulation of meristem development, and downregulated in *vrs5* throughout development compared to Bowman (Fig. 4B; Supplementary Fig. S4). Next to that, a VRS5 DAP-peak was found ∼7 kb upstream of the coding sequence of *CENTRORADIALIS*. HvCEN acts as a floral repressor and mutants show early flowering, shorter spikes, and less tillers at maturity (Comadran et al. 2012; Bi et al. 2019). To confirm the DAP-seq results, we performed an Electrophoretic mobility shift assay (EMSA) using probes of DNA sequences extracted from the DAP-peak that contained the VRS5 binding site GGGNCCCAC. We used the DAP-peaks identified within upstream regions of *HvCEN, MADS4*, and *SOC1-like*. The peak upstream of *HvCEN* contained 2 putative VRS5 binding sites, while in the peaks upstream of *MADS4* and *SOC1-like,* one was found. Our results showed that VRS5 can indeed bind to the core VRS5 binding motif GGGNCCCAC in these peaks (Fig. 4C). To further confirm the binding of VRS5 to the core motif GGGNCCCAC and its role in regulating target gene expression, we performed a dual-luciferase transactivation assay. Regions identified in the DAP peaks upstream of *HvCEN*, *MADS4*, and *SOC1-like* were cloned upstream of the luciferase reporter gene. Transactivation was then tested in *Nicotiana benthamiana* using VRS5 as the effector and Renilla luciferase as an internal control (Fig. 4D). Coexpression of VRS5 significantly increased luciferase expression for all 3 reporters when compared to the control (Fig. 4E). Our data thus confirms that VRS5 is able to bind to the selected upstream regions, and regulate the expression of the downstream target, in this case luciferase. While our RNA-seq data showed that *MADS4* expression is increased in the absence of VRS5—suggesting that VRS5 reduces *MADS4* expression—the transactivation assay showed an increase in *MADS4* expression when VRS5 was present (Fig. 4A–E). This suggests that the actual direction of regulation in vivo may be influenced by the presence of additional cofactors that are not present in the transactivation assay.

Taken together, our data shows that VRS5 binds to a core GGGNCCCA motif present upstream of genes involved in inflorescence development to regulate their expression.

### DAP-seq and RNA-seq reveal a conserved mode of action of VRS5

**Figure 5.**
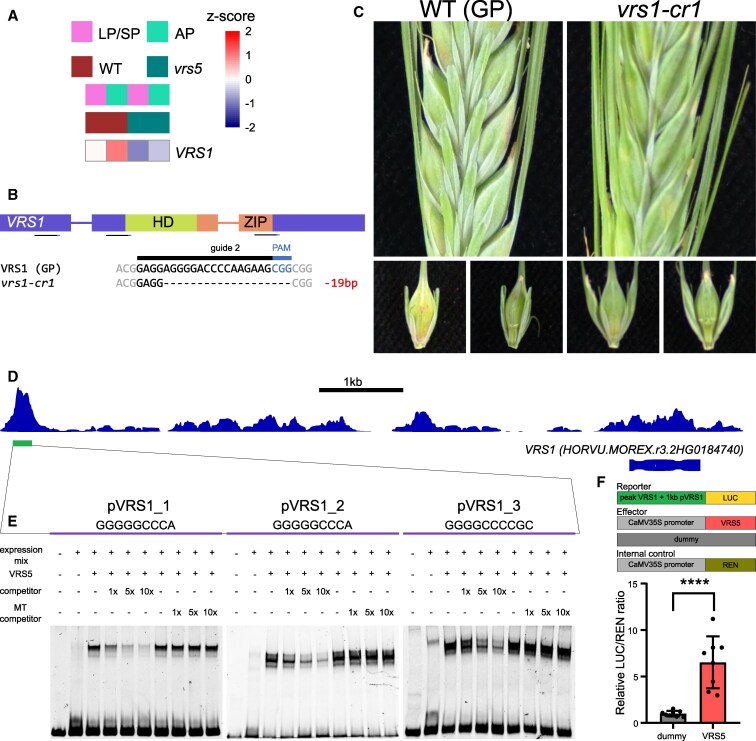
VRS5 binds to the promoter of VULGARE ROWTYPE SIX 1 (VRS1) to regulate its expression. **A)** Heatmap of z-scores of expression of VRS1 at developmental stages: LP/SP and awn primorida (AP) for wild-type (WT) and vrs5. Data are reanalyzed from GSE102191 (van Esse et al. 2017). **B)** Gene editing of vrs1 with targeted mutagenesis (CRISPR). **C)** Phenotype of gene edited vrs1 mutant in the genetic background of GOLDEN PROMISE (GP) compared to the GP parental line. VRS1 lines have 6-rowed spike architecture. **D)** VRS5 binding region identified with DAP-seq upstream of VRS1. There are 3 putative VRS5 binding sites within the VRS1 DAP-seq peak. **E)** EMSA confirms the DAP-seq data that demonstrated binding of VRS5 to the 3 VRS5 binding sites upstream of VRS1(pVRS1_1, pVRS1_2, and pVRS1_3). Each probe is ∼80 bp long and the sequence of the TCP binding site is indicated. For each probe, the first lane contains only labeled DNA probe, the second lane contains labeled DNA probe and expression mix without VRS5 plasmid, and the other lanes contain labeled DNA probe and expression mix with VRS5 plasmid. Unlabeled competitor or unlabeled shuffled competitor (MT competitor) is added to indicate lanes in 1×, 5×, and 10× concentrations compared to labeled probe. **F)** Dual-Luciferase transient transactivation assay. Data shows relative LUC/REN ratio in a dual-luciferase assay in *N. benthamiana* leaves using the DAP-peak identified upstream of VRS1 + 1 kb promoter of VRS1 in front of Luciferase. The mean LUC/REN ratios are relative to the mean LUC/REN ratio of the dummy. The red bar shows the mean LUC/REN ratio when VRS5 is used as effector. Black dots indicate individual measurements (*n* = 8). Error bars indicate standard deviation. Asterisks designate significant differences of the mean (Student's *t*-test, ****: *P* < 0.0001).

In the dicot model plant *Arabidopsis* BRC1, a homolog of *VRS5*, directly binds to the promoter of HD-Zip genes HB21, HB40, and HB53 to regulate their expression (Gonzalez-Grandio et al. 2017; van Es et al. 2023). The regulation of HD-Zip genes is conserved in maize, where ZmTB1 directly binds to the promoter of *GRASSY TILLERS* (*ZmGT1*). Given the conserved role of TB1 and BRC1 in regulation of HD-ZIP TFs, we first explored if among our bona fide targets HD-ZIP TF were present. Interestingly, the row-type related gene *VRS1* (*HORVU.MOREX.r3.2HG0184740*) was among the bona fide downstream targets of VRS5, showing a decreased expression at AP in *vrs5* compared to WT (Fig. 5A; Supplementary Table S7). *VRS1* encodes a HD-ZIP protein that is closely related to *ZmGT1* and the *Arabidopsis* HD-Zip genes. VRS1 is well-known for its role in lateral spikelet development in barley. Plants that do not have functional VRS1 exhibit 6-rowed spike architecture (Komatsuda et al. 2007; Liller et al. 2015; Zwirek et al. 2019). Our targeted mutagenesis using CRISPR corroborated the crucial role of *VRS1* in inhibiting the outgrowth of lateral florets (Fig. 5B and C). The VRS5 binding peak in our DAP-seq experiment was found around 7 kb upstream of the *VRS1* translational start site. Sequence analysis of the region of this DAP-peak showed that there are 3 putative VRS5 binding sites within the center of this peak (Fig. 5D). To verify if VRS5 was indeed capable of binding to the core VRS5 binding motifs found in this region, we performed an EMSA targeting all 3 putative VRS5 binding sites separately. This assay revealed that VRS5 was capable of binding to all 3 putative binding sites (Fig. 5E). Next to that, a dual-luciferase transactivation assay was done to show that VRS5 cannot only bind to this peak, but also use it to regulate Luciferase expression (Fig. 5F). Together, this provides evidence that VRS5 can directly bind upstream of *VRS1* to regulate *VRS1* expression.

Aside from *VRS1*, our transcriptional profiling combined with GO annotation showed that genes which are highly expressed at VA and low at the LP/SP stages (Group 1) are overrepresented for ABA and JA related genes. Previously, it was demonstrated in maize that ABA and JA related genes are among the putative downstream targets of ZmTB1 in tiller development (Dong et al. 2019). To further explore which bona fide VRS5 targets are conserved in maize, we used ZmTB1 targeted ABA and JA related genes and identified their orthologous genes in barley. All of the identified barley orthologs were downregulated in *vrs5* compared to WT and most were grouped in cluster Group 1A, based on their expression pattern (Supplementary Table S8). This suggests that these genes are mostly import early in development, when the tillers are formed. We found that indeed ZmTB1 and VRS5 share overlapping bona fide ABA and JA-related targets: AP2/B3 TF *RELATED TO ABI3/VP 2* (*RAV2*) (*HORVU.MOREX.r3.3HG0281730*); several lipoxygenases, including *TASSELSEED1* (*HORVU.MOREX.r3.2HG0171680*); and *JAZs* (Supplementary Table S8 and Fig. S4). This shows that the regulation of ABA and JA related genes is a conserved function between barley VRS5 and the maize ortholog TB1.

## Discussion

VRS5 is an important domestication gene in barley that plays a crucial role in tillering and row-type determination (Ramsay et al. 2011; Liller et al. 2015; Zwirek et al. 2019). Using a combination of transcriptomics and localizing genome-wide binding sites of VRS5, we identified its putative direct downstream targets. Different from ChIP-sequencing, which is a more in vivo method to find transcription factor binding sites and where inaccessible DNA cannot be bound, DAP-sequencing is a method where naked DNA is used, so chromatin context is not kept (Bartlett et al. 2017; Hajheidari and Huang 2022). Nevertheless, despite the lack of chromatin context in DAP-seq, the overlap between DAP-seq and ChIP-seq peaks is generally high (O’Malley et al. 2016). Of the DAP peaks found for VRS5, a significant part was found distal intergenic. A recent study performed DAP-sequencing of a large number of TFs in *Triticum urartu* that has a genome size comparable to barley. They found for the majority of TFs that more than 50% of the DAP-seq peaks were located more than 10 kb from the gene body (Zhang et al. 2021). Similarly, in strawberry DAP-seq of a TCP transcriptional regulator revealed also presence of the majority of peaks in the distal intergenic region (Chen et al. 2024). Together, this indicates that for DAP-sequencing on a large genome such as barley, a large portion of distal intergenic peaks can be expected. As an in vitro technique, DAP-sequencing might yield binding sites that would never be accessible under in vivo circumstances. In part, this could result in a higher number of distal intergenic DAP-peaks (Maher et al. 2018). To what extend distal intergenic DAP-peaks in general, and for VRS5 in this study, represent biological relevant long distal regulatory elements still needs to be elucidated. In barley, the most accessible chromatin regions (ACRs) are found between 10 and 100 kb from the nearest gene (Lu et al. 2019). Recent studies using STARR-seq indicate that about ∼37% of the enhancers had potential target genes at a distance of 10 kb, suggesting that only a minority of the genes in barley is regulated by adjacent enhancers (Zhou et al. 2024). In our study, we focused on DAP-peaks found within 15 kb of their nearest gene while a significant portion of the DAP-seq peaks was found in distal-intergenic regions. Given the observation that in barley, enhancers may lie up to 100 kb (Zhou et al. 2024) from a target gene, biological relevance of other DAP-peaks beyond our window cannot be excluded. Knowing the ACRs in tissues where VRS5 is expressed and under different environmental circumstances could give a better understanding and biological relevance to these other peaks. Taken together, to truly place distal-intergenic peaks into a biological context, a more in-depth study of the 3-D genome makeup at high resolution is imperative.

**Figure 6.**
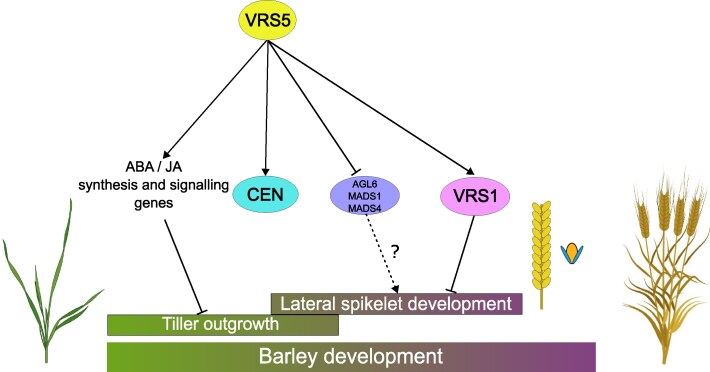
VRS5 network over development. VRS5 inhibits tiller outgrowth through regulating ABA/JA synthesis and signaling genes early in development. Later in development, VRS5 inhibits lateral spikelet development directly via VRS1. VRS5 also inhibits expression of AGL6, MADS1, and MADS4 directly, which might play a role in lateral spikelet development. Additionally, VRS5 induces the expression of HvCEN. On the right, a barley spike is shown next to a barley triple floret, where the central spikelet is shown in yellow and the 2 lateral spikelets in blue.

Nevertheless, in our analysis we identified >700 bona fide VRS5 targets and reveals an overlap in ABA and JA-related targets in maize and barley (Fig. 6). This suggests that the network of tiller development via ABA and JA signaling is partially conserved between barley and maize. In the model plant *Arabidopsis,* ABA related genes are directly targeted by the Class II TCP TF BRC1 to regulate axillary branching (Gonzalez-Grandio et al. 2017; van Es et al. 2023). This implies that the regulation of ABA signaling by BRC1/TB1 proteins is evolutionarily ancient and precedes the divergence of monocotyledonous and dicotyledonous plants (van Es et al. 2023). Interestingly, some of these genes found as bona fide VRS5 target may have a role in inflorescence development, from which the most known gene is *VRS1* (Fig. 6). Intriguingly, VRS1 and VRS5 alleles have been coselected for increased lateral grain size in 6-rowed cultivars (Ramsay et al. 2011), and *vrs5 vrs1* double mutants have an additive effect when compared to the respective single mutants (Zwirek et al. 2019). In addition, *vrs5* mutants have an intermediate phenotype, whereas *vrs1* lines have a complete 6-rowed phenotype (Liller et al. 2015; Zwirek et al. 2019). Combined, this suggests that there are other activators of *VRS1* that may act redundant, or in parallel to VRS5.

Aside from VRS1, our analysis also revealed other genes involved in inflorescence development among the bona fide VRS5 targets. For example, we identified a ortholog of *TASSELSEED1* as bona fide target of VRS5. In maize, *TASSELSEED1* is a lipoxygenase involved in JA biosynthesis and known for its important role in sex-determination of maize florets by repressing carpel development (Acosta et al. 2009). Furthermore, in our data we observed an upregulation of genes involved in flowering and floral organ identity, such as MIKC-type MADS-box genes, in *vrs5* compared to WT. MIKC-type MADS-box genes, which include the ABCDE-genes that are known for regulating floral homeotic patterning, are mostly upregulated during inflorescence development in barley (Digel et al. 2015; Liu et al. 2020; Kuijer et al. 2021). Our observation that floral homeotic genes are upregulated in *vrs5* when compared to Bowman may reflect an increased expression of these genes in lateral spikelet primordia in *vrs5*. However, the presence of a VRS5 DAP-peak within the promoter region of some of these MIKC-type MADS-box genes suggests a more direct effect of VRS5 on the regulation of these floral homeotic genes. Intriguingly, *HvMADS1*, which in barley regulates lemma and awn development and prevents inflorescence branching under high ambient temperatures (Li et al. 2021; Zhang et al. 2024a) is among the bona fide VRS5 targets. Moreover, an ortholog of AGL6 was identified as bona fide VRS5 target that is significantly upregulated in the *vrs5* mutant when compared to WT. In barley, *agl6* mutants are infertile due to defective pistils and homeotic conversions from 2 stamens into lemma-like structures in the central spikelets. In the lateral spikelets the underdeveloped stamens showed a homeotic conversion to bract-like structures in the *agl6* mutant (Shen et al. 2024). In wheat, *agl6* mutants are also infertile and TaAGL6 acts upstream of several other floral homeotic genes (Kong et al. 2022). These results suggest that VRS5 may also inhibit lateral spikelet development in 2-rowed cultivars by directly reducing the expression of these genes that are involved in floral organ development (Fig. 6). In agreement with this hypothesis, row-type genes prevent the development of carpels in the 2-rowed spike architecture (Zwirek et al. 2019), while loss of function mutants of *vrs5* and other row-type genes have an increased lateral floret fertility. While *ZmTASSELSEED1* was found as target of ZmTB1, orthologs of the other genes involved in floral development targeted by VRS5 were not found as target of ZmTB1 or AtBRC1. This implies a more specific group of targets for barley VRS5 in lateral spikelet development, which is a barley specific function of VRS5 compared to its orthologs, although differences due to sampling between our experiment and previous data sets generated on ZmTB1 and AtBRC1 (Dong et al. 2019; van Es et al. 2023) cannot be excluded. So next to a more conserved set of VRS5 targets in tillering a more barley specific set of targets is found in regulating lateral spikelet development.

Aside from the floral homeotic genes, *HvCEN* is also identified among the bona fide targets VRS5. HvCEN acts antagonistically to FLOWERING LOCUS-T (FT) in regulating floral transition (Bi et al. 2019; Colleoni et al. 2024). In wheat and *Arabidopsis*, TaTB-D1 and AtBRC1 respectively, are shown to interact with the FT1 ortholog of their species (Niwa et al. 2013; Dixon et al. 2020). To what extent binding of VRS5 to the *HvCEN* promoter impacts on floral transition, flowering or meristem development remains to be elucidated (Fig. 6). However, our results do suggest a role of VRS5 in the direct regulation of *PEBP* genes through binding to the promoter to activate the expression aside from the previously described regulation through protein–protein interaction.

In conclusion, with this study, we showed that barley VRS5 binds a class II TCP DNA motif GGGNCCCAC and acts mainly as a transcriptional activator. Bona fide VRS5 target genes include genes involved in ABA-signaling, which seems to be a conserved role for TB1-like proteins in both monocots and dicots to regulate tillering or axillary branching. We also revealed a role for VRS5 in regulation of floral homeotic and meristem identity genes. Our study exemplifies that it is essential to not only identify the molecular networks in model plants like *Arabidopsis*, but also in key crops such as barley to reveal conservation, but more important to identify divergence explaining the species-specific architecture. The study presented here offers a solid and crucial starting point for mutant analyses of a selection of bona fide targets to gain a deeper understanding of the VRS5 network and to enlighten its mode of action in shaping barley development.

## Materials and methods

### Phenotypic analysis

Seeds of Bowman and *int-c.5* (GSHO 2003) were stratified on wet tissue paper in the dark at 4 °C for 3 d, before sowing them in 1.3 L pots. Plants were grown in the greenhouse under 16 h light 21 °C, 8 h dark 18 °C conditions. Tiller number was counted per plant at 7, 10, and 14 d after emergence (DAE). The same procedure and growth conditions were used for Golden Promise, and *vrs1* mutants in this background. Pictures of the spikes were taken after flowering when the seeds were filling.

### RNA isolation and RNA-sequencing

Seeds of cv. Bowman and *int-c.5* were sown in 84-well trays. A stratification treatment by exposing the seeds at 4 °C in the dark for 3 d was provided to enhance equal germination. After stratification, plants were transferred to soil in a growth chamber under long day (LD) conditions (16 h light/8 h dark) at 20 °C. Developmental stages of the meristems were scored using the Waddington scale (WADDINGTON et al. 1983) by dissecting 3 plants of each genotype on the day of sampling. Shoot apical meristems were sampled over time at 4 different stages: W0, VA; W1, TA; W2.25, TM; and W3-3.5, LP/SP. Samples were taken in the afternoon (6 h before the end of the LD period). Leaves surrounding the apex were removed manually, before cutting the apex. Apexes were immediately frozen in liquid nitrogen and stored at −80 °C before RNA isolation. Three biological replicates were taken for each developmental stage, each consisting of 10–20 apexes. Frozen tissue was disrupted using the Tissuelyser LT (Qiagen). RNA extraction was performed on disrupted tissues using columns from the GeneJET plasmid miniprep kit (Thermo Fischer Scientific), as described previously (Yaffe et al. 2012). Extracted RNA was treated with DNase I (Qiagen) and RiboLock (Fermentas).

Extracted RNA samples that were used for RNA-seq were sent to Novogene Europe (Cambridge Science Park, United Kingdom), where mRNA was purified from total RNA using poly-T oligo-attached magnetic beads. After fragmentation, the first strand cDNA was synthesized using random hexamer primers, followed by the second strand cDNA synthesis. Samples were sequenced using the Illumina platform with a sequencing depth of 20 million reads per sample using 150 bp paired-end reads.

### Differential gene expression analysis

Raw paired RNA-seq reads were cleaned using Trimmomatic 0.39 (Bolger et al. 2014) with parameters: ILLUMINACLIP:adapters.fa:2:30:10 LEADING:3 TRAILING:3 SLIDINGWINDOW:4:15 MINLEN:36. The file adapters.fa contains nucleotide sequences of Illumina adapters used for library construction. From the trimmed reads, transcripts were quantified with the MorexV3 transcriptome as reference (Mascher et al. 2021) using salmon (Patro et al. 2017) and the softclip parameter (Supplementary Table S9). Differentially expressed genes were identified using the R package DESeq2 (Love et al. 2014) per developmental stage (VA, TA, TM, and LP/SP), and over development by adding developmental stage to the model (GLM). Identified differentially expressed genes were considered significant when the false discovery rate (FDR) < 0.05 and the absolute log2 fold change was more than 0.58. Data from the later developmental stages, namely the SP stage, and the AP stage, when the carpels are formed and the first phenotypical differences between Bowman and *int-c.5* in spike development emerge, are extracted from Gene Expression Omnibus (GEO) expression data set GSE102191 (van Esse et al. 2017). The data from these stages were reanalyzed against the Morex V3 genome in the same way as the newly generated RNA-seq samples from the VA, TA, TM, and LP/SP developmental stages (Supplementary Table S5). Data from the tiller buds specifically of *int-c.5* and Bowman were extracted from microarrays performed before (Wang et al. 2022). The differentially expressed genes between *int-c.5* and Bowman tiller buds under control conditions were reannotated to MorexV3 by blasting the sequences of the harvEST:Barley ID against MorexV3 (Supplementary Table S6).

### Quantitative-PCR

RNA from the above described samples was used to make first strand cDNA from 1 µg RNA per sample using iScript cDNA Synthesis Kit (Bio-Rad). qPCR was performed using iQ SYBR Green Supermix (Bio-Rad). Relative expression compared to *HvActin* is calculated (Gines et al. 2018). Primers used are described in Supplementary Table S10.

### Gene names, GO term annotation, enrichment, and cluster analysis

Unless previously characterized, gene names are based on the orthologous genes in rice according to ensemble plants release 60 (https://plants.ensembl.org/index.html). Naming of MIKCc MADS box genes is in accordance with Kuijer et al. 2021. Using Pannzer2 (Törönen et al. 2018), the coding sequences of MorexV3 reference genome (Mascher et al. 2021) were annotated and assigned GO-terms (accessed 21-06-2023). Genes that were significant differentially expressed in at least 1 developmental stage and/or independent of developmental stage were clustered based on their expression pattern per genotype using the degPatterns function from the R package DEGreport. In total, 2,869 transcripts show differential expression with |Log2FoldChange| > 0.58 and FDR < 0.05 in at least 1 of the developmental stages or independent of developmental time in the GLM. Clusters were grouped manually based on their global trend over time in 6 cluster groups. GO-term enrichment of the differentially expressed genes was done for each cluster group using the R package topGO using the “classic” algorithm as parameter followed by the Fisher's Exact Test, taking into account only those GO-terms that have at least 10 annotated genes in the designated term.

### DNA affinity purification and sequencing

DNA libraries for DAP-seq were made as previously described (Bartlett et al. 2017). For each replicate, genomic DNA was extracted from 100 mg developing inflorescences of barley cv. Morex, 4 weeks after sowing in 84-well trays (Waddington stage ∼W4) using the DNeasy Plant Mini kit (Qiagen). The DNA was fragmented with 10 sonication steps of 10 s using the Sonicator 150 (MSE) and allowing 45 s of rest on ice between each step. The fragmented DNA was end-repaired using the End-It kit (Lucigen) and A-tailed using Klenow fragment (3′-5′ exo-; NEB). The truncated Illumina Y-adapter was ligated to the DNA using T4 DNA ligase (Promega).

The CDS of VRS5 (cv. Bowman) without the stop codon was cloned, via Golden Gate cloning, together with a C-terminal 3xFLAGtag (MoClo toolbox; pICSL50007) (Engler et al. 2014) behind the SP6 promoter into the pSPUTK expression vector (Stratagene) that was made Golden Gate compatible as described (Kerstens et al. 2024). Similarly, eGFP was cloned from pICH41531. The FLAG-tagged VRS5 and GFP were expressed in vitro using 2 µg plasmid and TnT SP6 High-Yield Wheat Germ Protein Expression System (Promega). Expressed FLAG-tagged proteins were bound with prepared DNA libraries for 2 h at RT followed by immobilization by anti-FLAG magnetic beads (SIGMA). The mix was washed, and eventually DNA was eluted by competition with 3xFLAG peptide (APExBIO). Eluted DNA was amplified by PCR with primers completing the Illumina sequencing adapter of which 1 primer was indexed (Supplementary Table S10). Amplified products were run on a 1.5% agarose gel, and DNA between 200 and 600 bp was cut out and purified using GeneJET Gel Extraction Kit (Thermo Scientific). Six replicates (Supplementary Fig. S5) were sequenced using the HiSeq X-ten platform with a sequencing depth of 50 million reads per sample using 150 bp paired-end reads (BGI Europe).

### DAP-seq analysis

Raw paired DAP-seq reads were cleaned using Trimmomatic 0.39 (Bolger et al. 2014) using parameters ILLUMINACLIP:adapters.fa:2:30:10 LEADING:3 TRAILING:3 SLIDINGWINDOW:4:15 MINLEN:36, where adapters.fa is a file containing nucleotide sequences of the used adapters (Supplementary Table S10). Paired reads were mapped to the Barley MorexV3 reference genome (Mascher et al. 2021) using BWA-MEM v0.7.17 (Li and Durbin 2009, 2010). Peak calling was done using MACS2 v2.2.7.1 (Zhang et al. 2008), using the call-peak function, where the VRS5 samples were compared to the GFP control samples. The genome size parameter was set to 5.4e9, corresponding to the barley genome size. The “KEEP-DUP” parameter was set to “auto”, meaning that the MACS2 algorithm calculates the number of reads that are allowed to be identical, leading to a maximum of 2 identical reads. Genomic regions that had a high number of reads in the control samples (GFP-FLAG) were identified using the “greenscreen” method (Klasfeld et al. 2022). Called peaks overlapping with a “greenscreen” region were filtered out using bedtools intersect function (Quinlan and Hall 2010). Peaks with a *q* < 10^−10^ were kept and considered true peaks. The DNA sequences of the true peaks were used for de novo motif discovery using STREME from MEME-suite v5.4.1 (Bailey et al. 2015; Bailey 2021). The bdg files from MACS2 output were converted to tdf files for visualization using the Integrative Genomics Viewer v.2.11.9 (Robinson et al. 2011).

### Overlap with ZmTB1 targets

To check if previously identified ZmTB1 targets (Dong et al. 2019) are also targeted by VRS5, we used Orthofinder (Emms and Kelly 2019) to infer the barley orthologs of the ZmTB1 targets. As input the following proteomes are downloaded from Phytozome: *Arabidopsis thaliana* TAIR10 (Lamesch et al. 2012), *Brachypodium distachyon* v3 (Vogel et al. 2010), *Hordeum vulgare* (barley) MorexV3 (Mascher et al. 2021), *Lolium perenne* MPB_Lper_Kyuss_1697 (Frei et al. 2021), *Triticum aestivum* (wheat) IWGSC (ENA: GCA_900519105.1), *Oryza sativa* (rice) IRGSP 1.0 (Ouyang et al. 2007), and *Zea mays* (maize) AGPv3.31 (NCBI: GCF_000005005.1), which was used as reference by Dong et al. 2019.

### Electrophoretic mobility shift assay

EMSA was performed as described previously (Smaczniak et al. 2012). Briefly, DNA probes of around 50 bp are designed from genomic regions and corresponding primers were synthesized (Biolegio). Synthesized primers were annealed and obtained probes were cloned into pGEM-T. Subsequently, the probes were labeled using CY5.5 labeled primers using PCR based amplification. The VRS5 CDS was cloned into pDONR201 (Gateway), and subsequently, into a gateway compatible pSPUTK expression vector (Stratagene), which uses a SP6 promoter to induce expression (Jiang et al. 2022). The VRS5 protein was prepared in vitro using TNT SP6 Wheat germ protein expression system (Promega). Using the VRS5 protein, a EMSA binding reaction was performed for 45 min on ice using a binding reaction mixture (1.2 mm EDTA (pH 8.0), 0.25 mg/ml BSA, 7.2 mm HEPES (pH 7.3), 0.7 mm DTT, 60 μg/ml salmon sperm DNA, 1.3 mm spermidine, 2.5% CHAPS, 8% glycerol, 6.6 nmol/ml single-labeled dsDNA, and 2 μl of in vitro-synthesized proteins). When an unlabeled competitor probe was used, 6.6 nmol/ml (1×), 33 nmol/ml (5×), or 66 nmol/ml (10×) was added to the binding reaction mixture. The mixture was then loaded on a 5% polyacrylamide gel. The gel was run in 1× TBE buffer at 75 V for about 90 min. Labeled DNA shifts were visualized using a LiCor Odyssey imaging system at 700 nm.

### Dual-luciferase transactivation assay

To test transactivation of luciferase by VRS5 constructs were created using Golden Gate cloning (Engler et al. 2014). Synthesized DNA fragments containing identified DAP-peaks were synthesized and cloned in front of an minimal 35S promoter followed by Luciferase CDS terminated by a NOS terminator, using an in-house vector (available at Addgene: #231035), to form the expression vector. Next to that the CDS of VRS5 was cloned into pL0-CDS (pICH41308) and subsequently into pL1-F2 (pICH47742) behind a 2xCaMV 35S promoter (pICH51288) and terminated by a NOS terminator (pICH41421) to form the effector vector. A third pL1 construct was used to normalize luciferase activity. This control vector contains the Renilla Luciferase coding sequence, made Golden Gate compatible from pGreenII-0800 (Hellens et al. 2005), and a 2xCaMV 35S promoter and NOS terminator. For all combinations, the 3 L1 vectors were assembled in a pL2 (pAGM4723). As negative controls the effector plasmid was replaced by a pL1-F2 dummy (pICH54022). The assembled L2 constructs were transformed into *A. tumefaciens* strain AGL1 pSOUP. Transformed cells were grown overnight at 28 °C in LB. This culture was used to inoculate YEB containing 200 µM Acetosyrengone and 10 mm MES. This culture was grown overnight at 28 °C to an OD_600_ of ∼1. These cell cultures were spun down and resuspended in 15 mL MMA containing 200 µM Acetosyrengone and left at 28 °C for 2–3 h. For transfection, the abaxial side of leaves of 4–5 week old (nonflowering) *N. benthamiana* plants were infiltrated with the *A. tum* cell culture with an OD ∼1, using a needleless syringe. At least 5 different plants were infected with the same construct and were treated as biological replicates. The plants were grown for 2 d in a growth chamber. Samples of infiltrated leaf parts were taken and snap-frozen in liquid nitrogen. For luciferase measurement of the samples, the Dual-Luciferase Reporter Assay kit (Promega) was used. Measurements were done using a GlowMax Navigator Microplate Luminometer. For each sample the ratio of Firefly/Renilla Lucifarase was calculated relative to the mean ratio of each expression vector combination without VRS5 TF. Statistical difference was tested using Student's *t*-tests, for each expression vector.

### CRISPR-Cas9 mutagenesis

For CRISPR-Cas9 targeted mutagenesis of *VRS1*, 3 guides were designed, targeting different regions of the coding sequence. (guide 1: 5′-ACGTGGACACGACTTTCTTC-3′, guide 2: 5′-GAGGAGGGGACCCCAAGAAG-3′, and guide 3: 5′-GGAGGTGCGGCGCCTCAGGT-3′). Final vectors were created using both our in-house CRISPR vector system (available at Addgene: #231030 - #231033) and already available modular cloning toolkits (Engler et al. 2014; Lawrenson et al. 2015). Guides were synthesized as complementary oligos (Supplementary Table S10) and cloned in an optimized Cas9 scaffold (Dang et al. 2015; Parry et al. 2016) (scaffold sequence, where NNNNNNNNNNNNNNNNNNNN corresponds to the guide: 5′GNNNNNNNNNNNNNNNNNNNNGTTTCAGAGCTATGCTGGAAACAGCATAGCAAGTTGAAATAAGGCTAGTCCGTTATCAACTTGAAAAAGTGGCACCGAGTCGGTGCTTTTTTTCTAGACCCAGCTTTCTTGTACAAAGTTGGCATTACGCTAGAGACCACGAAGTG-3′). The level 1 backbone vector with the modified scaffold has been designed so that the digestion-ligation of the double-stranded guide using the Type IIS restriction enzyme BsmBI removes the mRFP1 expression cassette (Engler et al. 2014). This allows for a pink–white screening of the recombined level 1 vectors. The final level 2 vector used for plant transformation contains the intron-optimized hygromycin resistance cassette (pICSL11059), the Cas9 expression cassette (pICSL11056), and the 3 single guide RNAs targeting *VRS1* coding sequence under the control of the TaU6 promoter. The sequence-verified construct was introduced in the hypervirulent *Agrobacterium tumefaciens* strain *AGL1 pSOUP*, and used for *Agrobacterium*-mediated transformation of barley cv. *Golden Promise* immature embryos (Hensel et al. 2009). T_0_ plants were genotyped for CRISPR cuts using the Phire Plant Direct PCR Kit (Thermo Fisher Scientific) using primers described in Supplementary Table S10. Amplified fragments were sequenced directly.

### Accession numbers

Sequence data from this article can be found in the GenBank/EMBL data libraries under accession numbers_*VRS5:*  LT727721.1, *HvCEN:*  JX648191.1, *MADS4:*  AY541066.1, *SOC1-like:*  XM_045115699.1, *VRS1:*  AB489127.1.

## Supplementary Material

kiaf309_Supplementary_Data

## Data Availability

The data generated in this publication have been deposited in NCBI's Gene Expression Omnibus (Edgar et al. 2002; Barrett et al. 2012) and are accessible through GEO Series accession number GSE272367 and GSE272368.

## Supplementary data

The following materials are available in the online version of this article.

**Supplementary Figure S1.** VRS5 influences tillering and row-type.

**Supplementary Figure S2.** RNA-seq quality control.

**Supplementary Figure S3.** Grouping of coexpression clusters.

**Supplementary Figure S4.**q-PCR confirmation of RNA-seq data for a selection of targets.

**Supplementary Figure S5.** Experimental set-up for DAP-Seq of VRS5.

**Supplementary Table S1.** DESEQ2 results for each gene.

**Supplementary Table S2.** division of differentially expressed genes over expression cluster groups.

**Supplementary Table S3.** DAP-sequencing peaks of VRS5 and their location and annotation in the genome.

**Supplementary Table S4.** Distribution of annotation of DAP-sequencing peaks of VRS5 in the genome.

**Supplementary Table S5.** DESEQ2 results of the reanalysis of GSE102191.

**Supplementary Table S6.** Reannotation of Microarray of barley tiller buds of int-c.5 vs. WT published by Wang et al. (2022).

**Supplementary Table S7.** list of bona fide VRS5 targets.

**Supplementary Table S8.** list of ZmTB1 targets involved in Jasmonate (JA) and abscisic acid (ABA) synthesis and signaling and their corresponding barley orthologs.

**Supplementary Table S9.** TPM values for each gene for each sample.

**Supplementary Table S10.** Primers used in this study.

## Dive Curated Terms

The following phenotypic, genotypic, and functional terms are of significance to the work described in this paper:

BRC1 Gramene: AT3G18550
BRC1 Araport: AT3G18550

AGL6 Gramene: HORVU.MOREX.r3.6HG0604360
AGL6 Araport: HORVU.MOREX.r3.6HG0604360

RAV2 Gramene: HORVU.MOREX.r3.3HG0281730
RAV2 Araport: HORVU.MOREX.r3.3HG0281730

EMSA Gramene: electrophoretic mobility shift assay
EMSA Araport: electrophoretic mobility shift assay

NCBI Gramene: https://www.ncbi.nlm.nih.gov
NCBI Araport: https://www.ncbi.nlm.nih.gov

CENTRORADIALIS Gramene: JX648191.1
CENTRORADIALIS Araport: JX648191.1

VRS5 Gramene: LT727721.1,
VRS5 Araport: LT727721.1,

HvCEN Gramene: JX648191.1
HvCEN Araport: JX648191.1

MADS4 Gramene: AY541066.1
MADS4 Araport: AY541066.1

SOC1-like Gramene: XM_045115699.1
SOC1-like Araport: XM_045115699.1

VRS1 Gramene: AB489127.1.
VRS1 Araport: AB489127.1.
