# Supplementary material for: VULGARE ROW-TYPE SIX 5 binds to the promoter of tillering and floral homeotic genes to regulate their expression
Source: Plant Physiol. 2025 Jul 17;198(4):kiaf309. doi: 10.1093/plphys/kiaf309 (PMC12360928; doi:10.1093/plphys/kiaf309)
Supplement: kiaf309_Supplementary_Data [file kiaf309_supplementary_data.zip › Winkelmolen_etal_SupplementaryFigures.pdf]

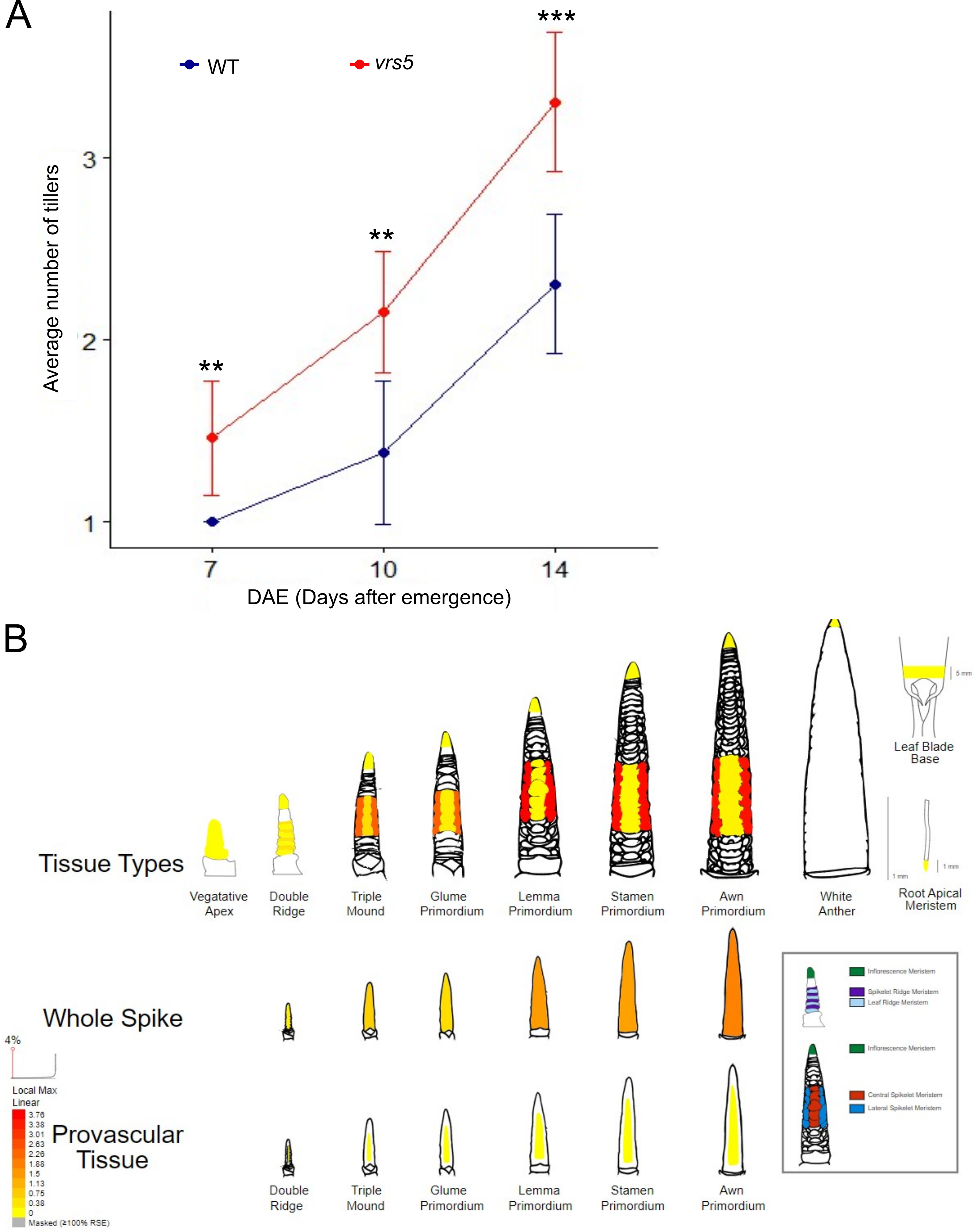

Supplementary Figure S1: VRS5 influences tillering and row-type. A) Line graph of mean number of *tillers per plant over time with int-c.5 shown in red and Bowman in blue. n=13 for each genotype. Differences at each timepoint are calculated using student's t-test; asterisks indicate significance (*: p < 0.05, **: p < 0.01, ***: p < 0.001). Error bars indicate standard error of the mean. B) expression pattern of VRS5 (HORVU.MOREX.r3.4HG0336690) retrieved from the ePlant expression atlas that uses the Thiel et al., 2021 dataset. Red indicates high expression, whereas yellow indicates low expression.*

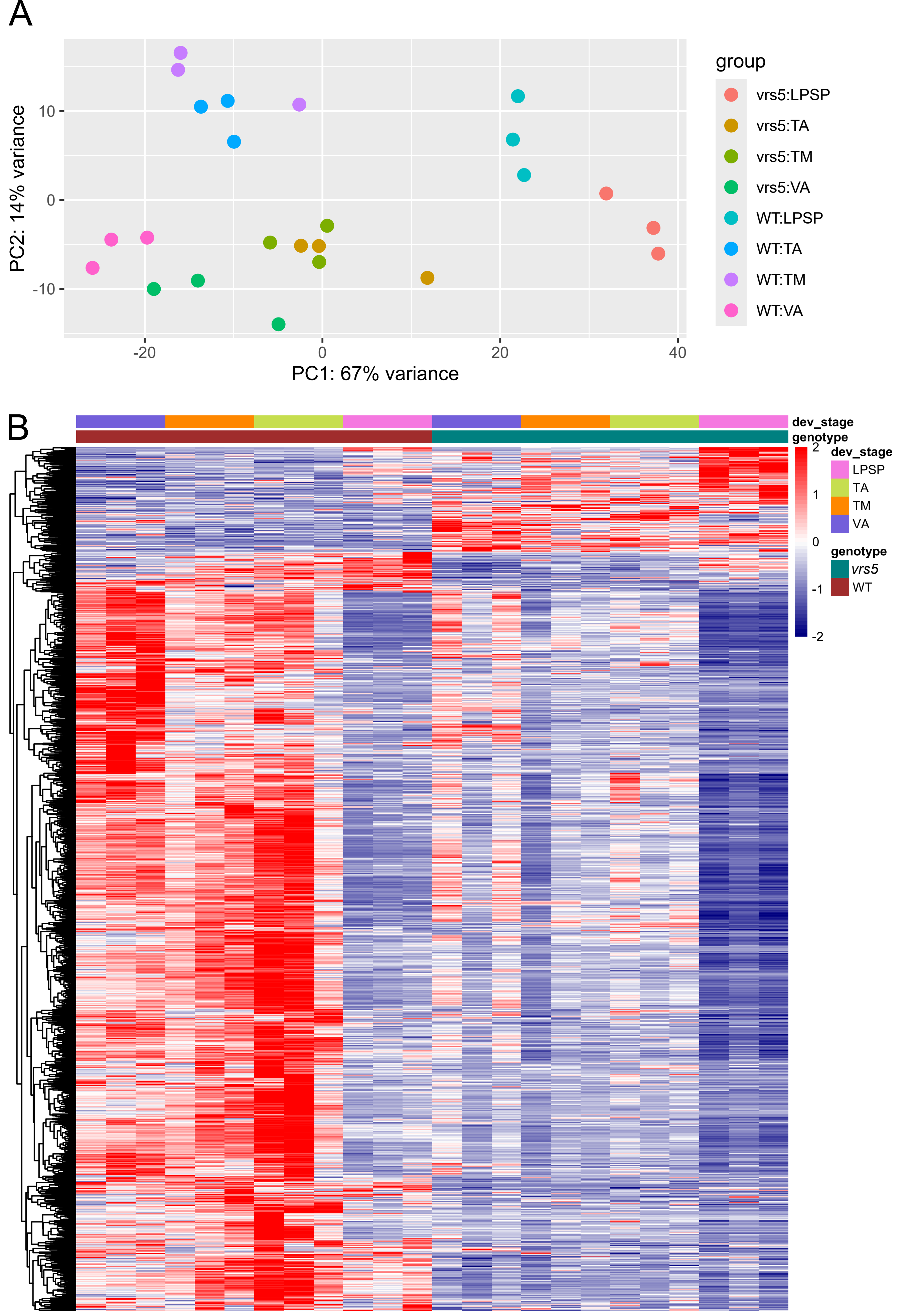

Supplementary Figure S2: RNA-seq quality control *A) Principal component analysis of the TPM values of RNA -seq samples. Dots indicated with the same color represents replicates of the same sample group. B) Clustered heatmap of z -scores per sample of expression levels of differentially expressed transcripts (DETs) between WT (Bowman) and vrs5 (int-c.5) over developmental stages tested, namely: vegetative apex (VA), transition apex (TA), triple mound (TM), and lemma and stamen primordia (LP/SP). For each developmental stage and genotype combination three biological replicates are used. The columns represent the different samples, grouped by genotype and developmental stage. Each row represents a DET.*

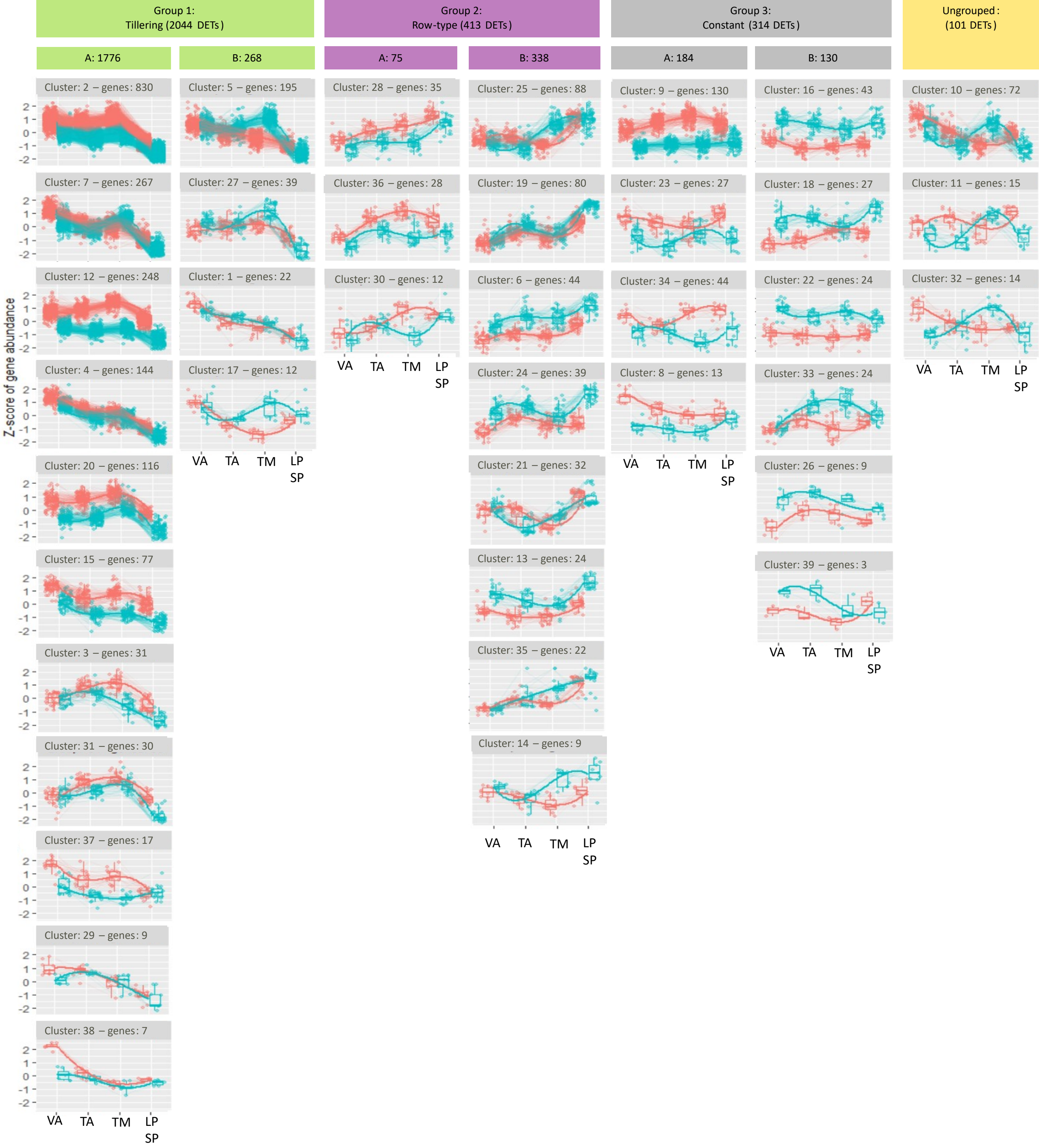

Supplementary Figure S3*: Grouping of co-expression clusters.* *Co-expression clusters of differentially expressed genes between vrs5 and WT over development using R package degPatterns, grouped in three main groups: 1) downward expression throughout development; 2) upward expression throughout development; 3) static expression throughout development; and lastly ungrouped clusters that do not follow either of the trends. The groups are sub-divided in A (lower expression in* vrs5 *compared to WT), and B (higher expression in* vrs5 compared to WT). *Each cluster shows the z-score of expression of the genes within the cluster, colored by genotype (red: WT (Bowman), cyan: vrs5 (int.c.5)) over developmental stages tested, namely: vegetative apex (VA), transition apex (TA), triple mound (TM), and lemma and stamen primordia (LP/SP). The boxes indicate median +/- quartile, the whiskers indicate mini mum and maximum values excluding outliers. For each developmental stage and genotype combination three biological replicates are used.*

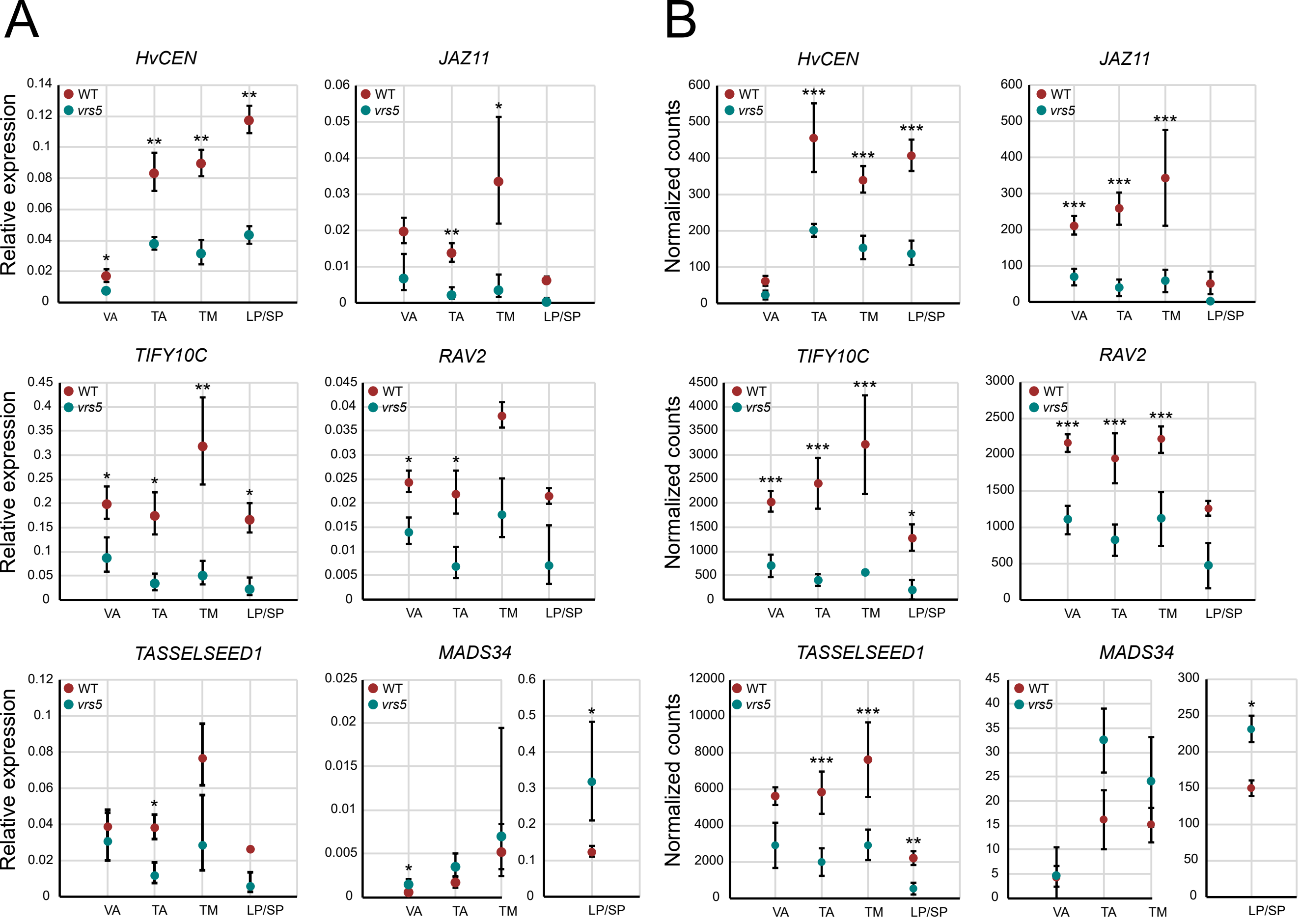

Supplementary Figure S4: q-PCR confirmation of RNA-seq data for a selection of targets *A) Relative expression of* HvCEN, JAZ11, TIFY10C, RAV2, TASSELSEED1, and MADS34 *compared to* Actin *using qRT-PCR and B) Normalized expression of* HvCEN, JAZ11, TIFY10C, RAV2, TASSELSEED1, and MADS34 *in our RNA-seq dataset at each developmental stage tested: vegetative apex (VA), transition apex (TA), triple mound (TM), and lemma and stamen primordia (LP/SP) in WT (red) and* vrs5 *(cyan). The error bars indicate standard deviation. For each developmental stage and genotype combination three biological replicates are used. Significance in A is tested using a Student's t-test at each developmental stage and indicated using asterisks (*: p< 0.05, **: p< 0.01, ***: p<0.001). Significance in B is tested using DESEQ2 and indicated using asterisks (*: FDR< 0.05, **: FDR< 0.01, ***: FDR<0.001).*

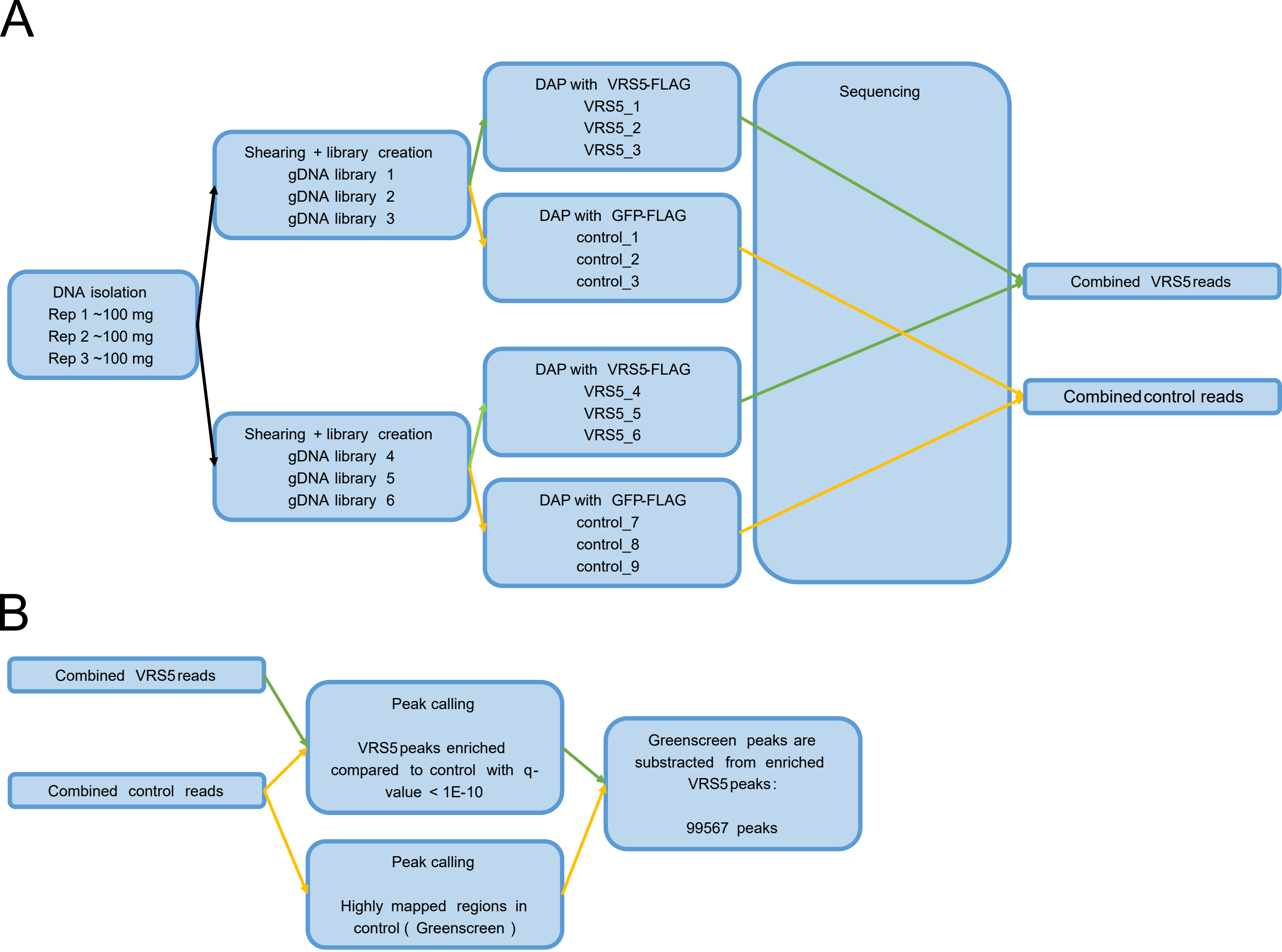

Supplementary Figure S5: Set-up DAP-Seq experiment VRS5. A) generation of DAP-Seq libraries that *are sequenced. B) Bioinformatic analysis scheme for the DAP-Seq libraries.*
